# A cross-species analysis of systemic mediators of repair and complex tissue regeneration

**DOI:** 10.1038/s41536-021-00130-6

**Published:** 2021-04-01

**Authors:** Julia Losner, Katharine Courtemanche, Jessica L. Whited

**Affiliations:** Department of Stem Cell and Regenerative Biology, Harvard University, Cambridge, MA USA

**Keywords:** Regeneration, Stem-cell research

## Abstract

Regeneration is an elegant and complex process informed by both local and long-range signals. Many current studies on regeneration are largely limited to investigations of local modulators within a canonical cohort of model organisms. Enhanced genetic tools increasingly enable precise temporal and spatial perturbations within these model regenerators, and these have primarily been applied to cells within the local injury site. Meanwhile, many aspects of broader spatial regulators of regeneration have not yet been examined with the same level of scrutiny. Recent studies have shed important insight into the significant effects of environmental cues and circulating factors on the regenerative process. These observations highlight that consideration of more systemic and possibly more broadly acting cues will also be critical to fully understand complex tissue regeneration. In this review, we explore the ways in which systemic cues and circulating factors affect the initiation of regeneration, the regenerative process, and its outcome. As this is a broad topic, we conceptually divide the factors based on their initial input as either external cues (for example, starvation and light/dark cycle) or internal cues (for example, hormones); however, all of these inputs ultimately lead to internal responses. We consider studies performed in a diverse set of organisms, including vertebrates and invertebrates. Through analysis of systemic mediators of regeneration, we argue that increased investigation of these “systemic factors” could reveal novel insights that may pave the way for a diverse set of therapeutic avenues.

## Introduction

Regeneration is a phenomenon prevalent within a wide variety of organisms found across the globe. Among these organisms, regenerative capacity is varied^1,2^. In the context of regeneration research, this provides a wealth of model organisms spanning many taxa with varying degrees of regenerative ability with which to study^3^.

Although major differences may exist between these organisms and their overt regenerative abilities, certain drivers and mediators of the regenerative response are conserved between organisms^4–7^. These commonalities perhaps have been most extensively characterized with regard to local regulation at the wound site^8^. However, cues persistent outside of the immediate injury area also play a major role in regulating the regenerative response. As we will discuss, many of these factors were initially characterized in landmark studies in the 1960s, 1970s, and 1980s^9–13^. Although many of these findings have since been elucidated in greater detail, some remain unexplored at the mechanistic level. Moreover, there is much variability across the depth of literature on different systemic subtopics.

We aim to engage both external and internal systemic cues in our discussion. Although these all provoke an internal response in the organism, they differ in the origin of their instigating signal. While internal, hormonal cues are triggered within the organism itself, many other cues, such as light exposure and seasonality, trigger internal changes only after external, environmental instigation.

In the past few decades, the development of new technologies has allowed for more precise investigation into regenerative phenomena. In addition, identification of commonalities in mechanisms and systemic factors across species and regenerative contexts has become increasingly accessible, as the depth of research in the regeneration space has expanded over time. Some of these factors have been explored across many species and have been shown to be conserved^5,6,14,15^. Others are either not conserved, have not yet been investigated in mammalian models, or have been implicated in mammalian repair but remain unexplored in other species, particularly those with the highest intrinsic regenerative tendencies. This review highlights some of the most deeply studied systemic mediators of regeneration, as well as some that have recently been identified or utilized within novel therapeutics. We also include many additional systemic factors that are intriguing candidates for deeper, cross-species analysis. Although it is impossible to know the full extent to which these factors are conserved across species, and thus the extent to which they might be useful in the search for human therapies, we posit that these factors should be studied across species in order to ascertain whether or not this is the case.

It is important to acknowledge the distinction between regeneration and repair; for the purpose of this review, we define regeneration as the complete regrowth of lost tissue, whereas repair refers to the imperfect restoration of damaged tissue that is already present. The scope of this review covers both regeneration and repair, as many factors are implicated in both processes. Mammals generally have more limited regenerative tendencies, so there is a much greater depth of mammalian-based research in the repair and wound-healing literature, as compared with the regeneration literature. We also discuss highly regenerative non-mammalian model organisms because their regenerative toolkit—while not necessarily endogenous to humans—may be compatible with mechanisms of human healing.

**Fig. 1:**
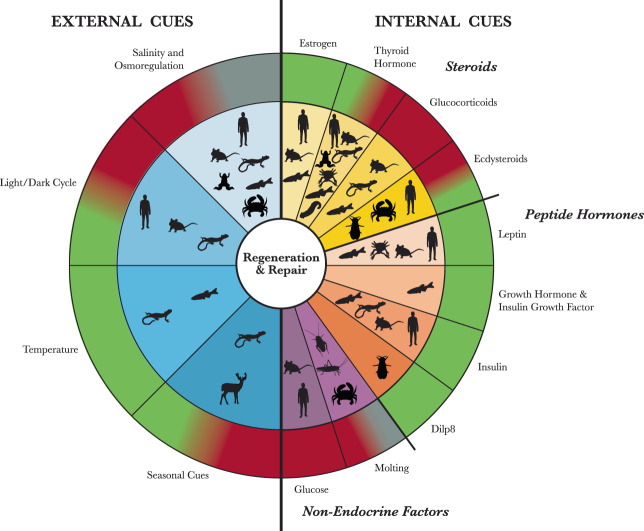
An overview of internal and external systemic mediators of regeneration and repair. Silhouettes indicate the organisms that will be discussed in the different sections of this review. The outer ring indicates the effect of increased application of each factor on the healing process; green indicates that an increase is beneficial for regeneration or repair, red indicates that an increase is detrimental to the healing process, and gray indicates a neutral or insignificant response. Gradients between colors within the same section indicate mixed results.

Our analysis of systemic factors in regeneration and wound healing will be divided into two sections (Fig. 1). First, we will discuss external factors, including salinity of the environment, temperature, and seasonal cues, among others. Second, we will explore internally derived, circulating factors, including glucose and several endocrine circulating factors. More specifically, we will discuss canonical growth factors such as growth hormone (GH) and insulin growth factor (IGF), as well as hormones less connoted with regeneration, such as estrogen, leptin, and insulin. Throughout, we provide examples from the literature, though an exhaustive coverage of all relevant findings is outside the scope possible here.

Studying regeneration and wound healing across many contexts allows for the discovery and acknowledgment of broad, conserved genetic, and physiological cascades, which would not be available in studies that are more limited in scope. This approach may also reveal differences in systemic influencers of repair and regeneration across species, which could present important clues to understanding varying regenerative tendencies. Consideration of these pathways, as well as contributions from non-mammalian-based research, could lead to novel insights that could be leveraged to help advance treatments for human healing.

## Eternal cues

### Salinity and osmoregulation

Many highly regenerative animals live in an aqueous or moist environment. Thus, one external cue to consider in the context of regeneration and repair is environmental salinity and its effect on the osmolarity and regenerative capacity of the organism. Consideration of the dietary salt intake of any organism may also be important in influencing regenerative success, although there are no current human studies explicitly considering dietary salt intake and regenerative capacity. The body responds to environmental and dietary salt content using a process called osmoregulation, which maintains the homeostatic concentrations of electrolytes in the blood, regardless of the environment^16^. These osmoregulators are found in the brain and kidney of most animals^16^.

One of the ways in which salinity has been shown to affect wound healing is in the induction of the inflammatory response in zebrafish^17^. Research shows that a gradient in osmolarity between the environment and interstitial fluid leads to the activation of cytosolic phospholipase a2 at the wound site, which works to recruit leukocytes to the injury^17^. This pro-inflammatory response and initial recruitment of leukocytes to the wound site propose an osmoregulatory mechanism for the induction of the healing process in zebrafish.

Osmoregulation has also been directly implicated in cellular responses to injury that are necessary for murine wound healing. For example, M2-activated macrophages are sensitive to osmotic conditions and have been demonstrated to use this input to guide efficient healing and repair responses in mice^18^. When fed high-salt diets, mice show reduced M2 activation following cutaneous injury and delayed wound healing compared with controls^18^. This same phenomenon also occurs in vitro, where increases in media Na+ concentrations of 10–40 µM cause cultured macrophages to show reduced signs of activation^18^. These experimental margins of increase were chosen to emulate the increase in sodium concentration in interstitial fluid due to a conventional high-salt diet, which is 40 µM^18^. Interestingly, this delay in wound healing occurs between 3 and 7 days post injury, during a highly proliferative phase of healing^18^. These results are similar to those from models with macrophage knockouts, although it cannot be directly concluded that the high-salt diet impairs wound healing by reducing M2 activation alone. Regardless, it is clear that this relationship between high salt and lower M2 macrophage activation regulates the wound healing process. A dependence on macrophage activity has also been proven in the axolotl, where successful macrophage infiltration of the regenerating structure is necessary for successful limb regeneration^19^. However, there is no published research regarding the ways in which variable salinity affects macrophages in the axolotl specifically, so the observed phenotype could be different from murine models.

A common consequence of osmoregulatory dysfunction in humans is hypertension, which can lead to skin ulceration and non-healing wounds of the extremities, particularly the lower legs^20^. The precise mechanism by which hypertension leads to problematic wound healing is unknown, although there are theories regarding failed keratinocyte migration and incessant inflammation^20^.

One important osmoregulator is the kidney, which produces a crucial protein called Renin. Renin, in addition to helping to maintain blood pressure, is a key player in fluid-electrolyte homeostasis^21^. Renin-producing cells retain some developmental plasticity and are highly involved in the regeneration of glomeruli in the kidney. The dysfunction of these renin-producing cells affects the regenerative ability of the kidney, demonstrating another link between normal osmoregulatory ability and regenerative capacity^22^. In addition, the tissue renin–angiotensin system, which has been traditionally studied as a regulator of blood pressure and blood sodium concentration^23^, is implicated in all stages of epidermal wound healing and repair^24^, as well as in the re-activation of infarct myofibroblasts after injury^25^.

The effect of variable environmental salinity on regeneration is clearly species- and context-dependent. For example, *Uca pugnax*, a species of crab native to estuaries, have adapted to thrive in an environment where the salinity varies both daily and seasonally^26^. When studied under four different salinity conditions, limb regeneration was invariable across these conditions, although increasing rates of mortality of the injured animals were observed in the highest salt concentrations^26^. The precise mechanism by which crabs regulate osmolality is still unknown. However, experiments investigating the reaction of the mud crab, *Scylla paramamosain*, to sudden drops in salinity have identified 135 differentially expressed genes between conditions, including a gene involved in the regulation of the renin–angiotensin system^27^. It is important to note that although research across 14 species of fiddler crab has shown that osmoregulatory ability in crabs likely evolved from varying salinity^28^, research on environmental salt concentration and regeneration in animals that have not adapted to thrive in variable salinity environments has not been published.

In addition, osmoregulation in aquatic animals, such as salamanders and froglets, relies on the nature of the barrier that the skin provides. Amphibians have fewer cornified layers of differentiated, dead cells to act as a barrier to the outside world, and several aspects of the general structure of the skin are consistent between humans and amphibians^29^. Although humans and other mammals do not live in aquatic environments, it is important to consider differences in the way these organisms—and their cells—are exposed to varying levels of salinity, whether through aquatic environments or through diet.

Physiological osmoregulators work in response to the salinity of both diet and environment. Many of the studies and processes outlined above regard unique biological processes environmental conditions, which may not pertain to humans or current therapeutic approaches. However, research considering both salinity of diet and environment must be expanded upon before it is possible to determine whether they could affect human wound-healing processes or downstream repair and regeneration events.

### Temperature

Temperature impacts all biological processes, notably through influence over biochemical reaction kinetics and enzyme activity. These temperature-induced modulations at the molecular level play a critical role in macro-level functions, including regeneration. Typically, warmer temperatures are associated with faster regeneration rates. For example, in a variety of fish species, rates of wound healing are proportional to ambient temperature, and this increased healing speed is correlated with faster rates of cellular processes, including macrophage activity and keratocyte migration to the wound site^30–33^. This effect has been observed even at temperatures above normal homeostatic range^32^. However, it is unclear whether temperature alone directly prompts these regenerative changes, as other temperature-dependent factors, such as metabolic rate^34^, may more directly prompt these changes in regenerative speed.

This pattern of faster regeneration at warmer temperatures may influence animal behavior. When housed in a thermal gradient apparatus, red-spotted newts undergoing limb regeneration display a preference for warmer temperatures of 24–25°C, ~2°C higher than the temperature preferences of their uninjured counterparts^35^. This preference may occur because 25°C has been associated with promoting maximal new longitudinal growth of the regenerating limb^11^. However, when considering differentiation in addition to outgrowth, the optimal temperature for maximal complete limb regeneration actually appears to be ~29°C^35^. Therefore, 24–25 °C preferred temperature of regenerating newts suggests that, although regeneration rate may increase with temperature, other biological processes may be negatively affected by temperature extremes, causing the desired temperature for habitation to be lower than what is most optimal for the regenerative process alone. Indeed, this relationship between regeneration and temperature preference may not be directly causal; further evidence is needed to determine the exact motivator of this preference.

Regardless of precise temperature preference, regenerating newts are more consistent in their external temperature preferences than those that are not undergoing regeneration^35^. This reduced temperature variability suggests that these ectotherms may require increased thermoregulatory control during regeneration, underscoring the importance of this external factor during the regenerative process.

It remains to be determined whether insights into the connections between thermoregulation and tissue regeneration in poikilotherms (organisms whose body temperature changes with their environment) might be relevant to humans. Although more research is needed to reveal the role that temperature might play in humans and other homeotherms (organisms that can regulate their body temperature to be different from that of their environment), there is indeed evidence that temperature is associated with regeneration in these organisms. Hirose et al.^36^ found an inverse relationship between the percentage of diploid cardiomyocytes-a proxy for heart regenerative capacity-present in various mammals and the respective body temperatures of those mammals. This correlation suggests that temperature-dependent mechanisms may influence regeneration in mammals, although the degree to which temperature actively influences regenerative ability is unclear.

### Light/dark cycle

Although commonly associated with sleep–wake cycles, circadian rhythms affect many diverse biological processes, including regeneration^37^. Light exposure has an important role in regulating the circadian cycle, and it consequently affects the systemic context in which regeneration occurs. Light and darkness provide external signals which, when translated by the pineal gland into day/night cycles, promote distinct hormonal and neural responses^38^. Some of the resulting modulations in regenerative capacity may be the direct result of light fluctuations, or they may be an indirect consequence of alterations to other light/dark cycle-dependent attributes, such as activity and sleep–wake cycles.

In newts, increased duration of light exposure appears to have positive effects on regenerative rate, with continuous light exposure prompting the fastest regenerative rate compared with other permutations of light and darkness^39^. This correlation is apparent even when the animals used are blind^39^. This phenomenon is speculated to be caused by activity within the pineal gland. When stimulated by light, the pineal gland produces serotonin, which stimulates prolactin release^40^. Generally, regeneration of complex, multi-tissue structures is facilitated by the transient development of a collection of activated progenitor cells near the injury site; this structure is called a blastema. The rate of blastema production and growth is a determinant of the overall observable rate of early regeneration in fins and limbs and may also be reflected in the rate of regeneration of the full structure in some instances. In these newts, serotonin-stimulated prolactin release promotes increased cell density at the blastema site, which may be due to increased cellular proliferation, decreased cell death, and/or increased blastema cell recruitment, and subsequently accelerates regeneration rate^10,40,41^. However, the precise molecular mechanism of prolactin action in the context of regeneration remains unknown.

Light exposure may also have a direct role in mammalian healing; however, unlike in salamanders, mammals appear to heal most effectively when circadian norms are unperturbed. For example, in a study of mouse corneal regeneration, disruption of the normal circadian cycle through alterations in light/dark exposure—including constant light, constant darkness, and a flipped light/dark cycle compared with environmental norms—had a negative impact on corneal epithelial mitosis and consequently impaired corneal repair after wounding^42^. This regeneration-hindering result prevailed even when the mice were subjected to constant light^42^. Similarly, long-term circadian rhythm dysregulation hinders the regenerative ability of skin and hair precursor cells in humans, as observed in a study comparing cultured skin biopsies of night-shift workers to diurnal workers^43^. These findings all bolster the claim that circadian perturbations have a strong impact on regenerative response, although the specific impact is variable between species and tissues.

Regardless of time of injury, several studies in mice have found that cellular proliferation rates follow a cyclic pattern during regeneration that corresponds with circadian oscillations, with the largest spike in proliferation typically occurring in the morning^42,44,45^. BMAL1, a circadian clock trans-activator that promotes stem cell self-renewal and negatively regulates replicative senescence^46^, has a key role in this response through the promotion of cell proliferation at the wound site^44,47^. Indeed, mice lacking BMAL1 expression display significant defects and lags in skin wound closure^48^.

BMAL1 may also be influential in wound healing because it causes oscillations in levels of actin regulators; the consequential modulation in actin activity causes fibroblast migration to the wound site to occur more rapidly for wounds inflicted during hours of activity compared with those incurred during times of rest^45^. This timeline replicates that of burn wound healing in humans, where burns incurred in the evening take ~60% longer to heal than those incurred during the day^45^.

In addition to its regulatory effect on circadian rhythm, light/dark cycles also modulate glucocorticoid rhythm. Although the role of glucocorticoids in regeneration has been studied (see “Glucocorticoids” subsection), the influence of light-dependent glucocorticoid modulations has yet to be explored and is an enticing topic that could advance regenerative biology.

### Seasonal cues

Seasonal cues can also play an influential role in the regenerative response, working in tandem with related factors such as age, developmental stage, sex, and reproductive cycle. Like circadian rhythms, seasonal cues are associated with cyclic cycles, although these have yearly periods as opposed to daily, circadian-associated turnover. Such factors may include annual fluctuations in daylight, temperature, humidity, diet, and microbiome, and they may trigger internal, hormonal changes that influence regeneration. Newts, for example, regenerate faster in the summer than in autumn or winter, even in conditions where light exposure and temperature are kept constant year-round^40,49^. This regenerative difference may be owing to seasonal variations in prolactin levels^50^; however, the overt seasonal signal remains unclear.

The influence of seasonal cues is better understood in deer. Deer antlers serve as an especially informative case study, as they are the only mammalian appendages that can completely and repeatedly regenerate^51^. While many other instances colloquially referred to as mammalian regeneration might be more appropriately considered repair processes, deer antlers are capable of true regeneration. Although they may visually appear similar to hair or fingernails when viewed from afar, growing antlers are complex, stem-cell derived, living organs composed of bone, vasculature, and nerves, enclosed by a unique epidermal covering known as velvet skin^52^. Each spring, male deer shed and regrow their antlers in preparation for the autumn mating season^53^, a cycle that is triggered by changing photoperiods^12,54^. The lengthening number of daylight hours in the spring prompts a variety of endocrine changes, implicating thyroid hormone, luteinizing hormone, testosterone, and parathyroid hormone^55–57^. Proteins that mediate the activity or metabolism of these hormones and the antler regenerative process overall have recently been identified through preliminary proteomics assays; these include Heat shock protein 90, which is involved in steroid hormone signaling, and mitogen-activated protein kinase-3, a regulator of stem cell activation and proliferation^58,59^. Further validation and functional assessments are needed to discern the precise roles of these proteins in antler regeneration, and it remains to be determined whether the deer antler regenerative is generalizable to other kinds of repair mechanisms in other tissue types and organisms.

## Internal cues

### Steroids: thyroid hormone

Thyroid hormone is one of the longest-studied hormones in the context of aging and regeneration^60^. Although often associated with metabolism, thyroid hormone also influences metamorphosis and regeneration. In urodeles (salamanders) and anurans (frogs), increased levels of thyroid hormone are required for metamorphosis^61^. Although naturally metamorphosing species of salamander retain their regenerative abilities^62^, thyroxine-induced metamorphosis in axolotls—a highly regenerative species of salamander that ordinarily remains in juvenile form throughout its life—leads to increased incidence of patterning defects and decreased overall rate of the regenerative process^63^. This response to metamorphosis is somewhat reminiscent of the transition that occurs in naturally-metamorphosing anurans, which fail to properly regenerate post-metamorphosis^64^. This transition illuminates the impact that thyroid hormone-induced metamorphosis may exert on regeneration.

Thyroid hormone has been implicated in the regeneration of a variety of physiological structures, including appendages, heart, and nerves. The role of thyroid hormone in regeneration of peripheral nerves was recently reviewed extensively^65^ and will not be discussed here. However, it is important to consider this relationship as a possible explanatory mechanism in studies linking thyroid hormone to complex tissue regeneration.

Thyroid hormone administration to cultured tadpole tails causes regression in tail size and increased expression of glucocorticoid receptors^66^, and increased glucocorticoid receptor expression is linked to a reduction in cell proliferation and is thus indicative of regenerative delays (see “Glucocorticoids” subsection). Exogenous thyroid hormone has a similar inhibitory effect on zebrafish heart regeneration^36^, whereas inactivation of thyroid hormone signaling in mice increases heart regenerative potential and proliferative activity^36^. Based on these findings, one might be compelled to conclude that regeneration is hindered by increased thyroid hormone and expedited by inhibition of thyroid hormone; however, this finding is not replicated in all organisms. For example, *Xenopus laevis* display impaired heart regeneration whenever thyroid hormone levels are significantly perturbed; this includes both when thyroid hormone signaling is inhibited and when it is overexpressed^67^. Moreover, although thyroid hormone-induced metamorphosis may interfere with regeneration in axolotls, other salamanders that undergo natural thyroid hormone-mediated metamorphosis, such as newts, retain full regenerative capabilities during adulthood^68^. Thus, differential responses to thyroid hormone signaling should be carefully considered when drawing connections between different organisms and regenerative contexts.

While thyroid hormone may be regulated differently in mammals than in amphibians, studies of hypothyroidism and hyperthyroidism have demonstrated that thyroid hormone nonetheless plays a role in mammalian wound healing. Hypothyroidism is most often associated with increased healing complications in both animal models and in humans^69^, although there is disagreement about whether this association with wound healing complications occurs among thyroxine-supplemented hypothyroid patients^70,71^. Discrepancies over whether hypothyroidism and thyroxine-supplementation influence wound healing may be resolved through repeated studies with increased sample sizes. In addition, the variance of surgical procedures undergone by patients between the different studies may also provide an explanation for conflicting results.

Meanwhile, studies pertaining to hyperthyroidism in mammals have indicated that increased levels of thyroid hormone are associated with improved cardiac regeneration outcomes. More specifically, this association between hyperthyroidism and accelerated wound healing has been demonstrated in rat cardiac tissue after myocardial infarction^72^. More recently, a report in mice provided tantalizing evidence that the thyroid hormone signaling system might indeed provide a productive therapeutic target for regenerative responses in the heart^36^. When thyroid hormone signaling was attenuated in adult mouse cardiomyocytes by expression of a dominant-negative thyroid hormone receptor, an increase in cardiomyocyte proliferation and reduced fibrosis were observed following cardiac injury^36^. Future work in humans may similarly uncover roles for thyroid hormone signaling in complex tissue regeneration.

Investigations on the influence of thyroid hormone on wound healing in human cells and tissues have been limited. In cultured human keratinocytes, exogenous thyroid hormone has been observed to stimulate expression of proliferation-associated keratin genes^73^; however, further investigations are needed to conclusively determine the endogenous role—if any—that thyroid hormone has in human wound healing.

### Steroids: glucocorticoids

Secreted by the adrenal cortex, corticosterone is a physiological glucocorticoid that is involved in various biological processes^74,75^. This steroid was first investigated in the context of regeneration owing to its involvement in stress response and inflammation^74,75^. Recent work has explored the relationship between corticosterone and regeneration in various physiological structures and in a variety of model organisms. For example, in Allegheny Mountain dusky salamanders, administration of ectopic corticosterone causes delays in tail regeneration^76^. Exogenous corticosterone treatment has also been shown to delay cutaneous wound healing in Allegheny Mountain dusky salamanders by interfering with the inflammatory process^77^, so it is plausible that the reported delays in tail regeneration may be caused by a similar inflammatory mechanism. Meanwhile, in fetal mouse cardiomyocytes, two recent studies have demonstrated that the administration of corticosterone results in a decrease in cell proliferation in vitro and in vivo^78^. In addition, cytokinesis inhibition was observed in cardiomyocytes harvested from postnatal day 1 mice and grown in culture^78^, although it was not observed during a separate in vivo study at postnatal day 7^79^, a difference that may be attributed to differences in the ages of the mice or to different environmental signals. Meanwhile, prohibiting corticosterone signaling through cardiomyocyte-specific glucocorticoid receptor ablation results in increased cardiomyocyte proliferation and heart regeneration after myocardial infarction at postnatal day 7^78^, although this result was not replicated in a study that treated mice with a glucocorticoid receptor antagonist after myocardial infarction at postnatal day 1^79^. Similarly to the ectopic corticosterone experiments in salamanders, the discrepancy between these findings may be the result of comparing experiments that used mice at two different ages.

Of note, although salamanders, rodents, birds, reptiles, and most other vertebrates use corticosterone as their primary physiological glucocorticoid, humans and some other mammals rely most heavily on cortisol, although corticosterone is also synthesized^80^. Thus, the aforementioned experiments, all performed in organisms that rely on corticosterone and do not synthesize cortisol, may not fully capture glucocorticoid-implicated healing mechanisms that are completely identical to those in humans.

Zebrafish are a useful organism for studying glucocorticoid effects that may be applicable to humans, as their primary stress hormone is cortisol^81^. Zebrafish treated with cortisol during embryogenesis experience chronically elevated cortisol levels during adulthood, leading to chronic inflammation^82^. These zebrafish also display defective tail fin regeneration, indicating that the role of cortisol and possibly other glucocorticoids in regeneration may be related to immunoregulatory and inflammatory responses^82^. Further investigation is needed in order to uncover the precise molecular mechanisms of glucocorticoid action during regeneration. Moreover, it is plausible that future studies may uncover additional, local effects of cortisol on other key components of the regenerative process, such as differentiation state and proliferation, within the blastema of the stem cells that give rise to the regenerated tissue.

### Steroids: estrogen

While perhaps best known for its influence on female primary and secondary sex characteristics, estrogen also has a prominent role in regeneration. This hormone promotes healing in various regenerative contexts, including cutaneous wound healing;^83–85^ skeletal muscle, bone, and cartilage regeneration^86–88^; and liver regeneration^89–91^.

Recent work on heart regeneration further underscores the influence of estrogen on healing processes. One study in female zebrafish demonstrated that injury to the heart increases plasma levels of estrogen and the expression of estrogen receptors^92^. The same study also found that treatment of male zebrafish with estrogen accelerated heart regeneration through the enhancement of immune and inflammatory responses^92^. By examining male and female zebrafish independently, the study accounts for an important factor in estrogen regeneration research: the differences in estrogen expression based on sex. In zebrafish, this sexual dimorphism has also been observed in pectoral fin regeneration, as increased androgen signaling and decreased glycogen synthase kinase-3 signaling in males leads to pectoral fin regenerative impairments^93^. Since physiological levels of estrogen (and other sex hormones) are differentially expressed in females versus males, experiments on the influence of estrogen on regeneration may yield different results depending on the sexual makeup of the experimental group^94–96^. These differences should be considered when designing experiments, drawing conclusions, and determining the therapeutic potential of estrogen-related regenerative findings.

Roles for estrogen in human healing are being elucidated. A recent heart regeneration study, in which human mesenchymal stem cells were co-cultured with murine heart slices, revealed that the addition of exogenous estrogen increases secretion of angiogenic factors by the human mesenchymal stem cells and prompts cell migration and incorporation into heart slices^97^; however, in vivo studies are needed to determine the clinical relevance of this result. In general, studies in humans repeatedly demonstrate that reduced estrogen causes defects in wound healing, while exogenous estrogen may promote improved healing outcomes^98^. These links between estrogen and human healing provide optimism that the estrogen-implicated regeneration pathways in regenerative model organisms may reveal methods to achieve increased efficiency in human healing.

### Steroids: ecdysteroids

Ecdysteroids are arthropod-specific steroid hormones. In crabs, endogenous ecdysteroids play an integral role in regulating reproduction, molting (see “Molting” subsection), and regeneration^99,100^. The relationship between crab ecdysteroids and regeneration is most clearly demonstrated in an ecdysteroid receptor RNAi knockdown experiment in fiddler crabs in which local injection of *ecdysone receptor* and *retinoid X receptor* dsRNA prevented blastema formation and inhibited cell proliferation^101^. However, ecdysteroids do not have a consistent effect on regeneration across all arthropod orders. Although ecdysone promotes *Drosophila* imaginal disc growth during larval development, upon injury, *Drosophelia* Insulin-Like Peptide 8-mediated downregulation of ecdysone is critical in order to slow down imaginal disc growth and allow for repair (see “Dilp8” subsection)^102^. During the third larval stage, ecdysone signaling underlies the closure of the window of regenerative potential, as increased ecdysone signaling turns off expression of *chinmo*, which encodes a transcription factor that maintains epithelial progenitors in an undifferentiated state and is required for imaginal disc regeneration, and initiates expression of *broad*, which encodes a transcription factor that creates a differentiation-permissive state^103^.

Although humans and other mammals do not produce ecdysteroids, exogenous ecdysteroid administration has been associated with the promotion of human keratinocyte differentiation and acceleration of skin wound repair^104^. This is not the only instance of an invertebrate-exclusive substance facilitating mammalian healing. Another invertebrate-specific factor, honeybee royal jelly, has recently been shown to improve the mammalian healing process, with in vitro effects on human keratinocytes parallel that of ecdysteroids^104,105^. In an in vitro model of human scratch wounds, administration of a water-soluble protein fraction derived from royal jelly resulted in faster wound closure accompanied by an increase in human epidermal keratinocyte proliferation and migration^105^. This result has translational relevance to human healing in vivo. Recently, a clinical study found that an ointment containing royal jelly proteins and panthenol significantly improved the healing of limb-threatening, diabetic foot wounds^106^. These findings demonstrate the potential utility of invertebrate regenerative mechanisms on human healing and bolster the potential for future ecdysteroid-based therapeutics for human wound repair.

### Peptide hormones: leptin

Some of the most deeply studied peptide hormones are those that control appetite; among these is leptin^107^. More recently, leptin has been implicated in tissue regeneration^108–111^. In the regenerating zebrafish heart and fin, epigenetic profiling has shown that there is an enhancer element for *lepb*, the zebrafish leptin homolog, which acquires open chromatin marks during regeneration^112^. This increased control over *lepb* transcription allows for injury-dependent expression^112^. This enhancer element, when transgenically manipulated to affect the expression of either pro- or anti-regenerative alleles, is able to dramatically improve or diminish regenerative outcomes^112^. In addition, inserting transgenic copies of this enhancer element activates *lepb* expression in injured neonatal mice^112^.

Leptin has been implicated in regeneration studies across many species. It is upregulated in the axolotl limb blastema^113^ and has been implicated in normal skin wound healing in mice^114^. Leptin-deficient mice show extremely delayed skin wound healing, which is rescued to the normal phenotype by either topical or systemic application of leptin^114^. This indicates that leptin is necessary for normal epidermal wound healing, although delayed wound healing can occur in the absence of leptin. Moreover, in wild-type mice, topically applied leptin improves the rate of healing^114^. Immunohistochemistry data show that wound-adjacent keratinocytes express leptin receptors, and in vitro studies demonstrate that both primary human keratinocytes and a keratinocyte cell line display increased mitosis when exposed to exogenous leptin^114^. In addition, fate-mapping experiments in mice have revealed that the main source of bone after injury is from leptin receptor-expressing mesenchymal stromal cells in the bone marrow^108^. However, a precise mechanism for the role of leptin in regenerative, rather than reparative processes in mammals has not yet been demonstrated.

### Peptide hormones: GrH and IGF

In addition to unexpected, regulatory hormones like leptin, several traditional growth hormones (GHs) are implicated in regenerative processes. Insulin growth factor (IGF) is required for zebrafish fin regeneration^115^. After amputation, a wound epidermis and blastema form, and these structures intercommunicate through IGF2-β signaling^115^. When IGF activity is inhibited, fin regeneration is impaired; apoptosis increases in the wound epidermis, proliferation of blastema cells decreases, and key markers of both cell types are lost^115^.

GH is a common regulator of IGFs in the liver as well as many other tissues. The addition of exogenous GH increases the rate of regeneration of zebrafish hair cells after acoustic trauma. This effect is mediated through greater cell proliferation and reduced apoptosis. Injection of a GH antagonist causes decreased cell proliferation and greater rates of apoptosis^116^. Because GH regulates IGFs, it is possible that this result is partially due to a subsequent upregulation of IGF, which has been shown to increase proliferation in cardiac regenerative events in the zebrafish^117^.

### Peptide hormones: insulin

Insulin is a peptide hormone produced by pancreatic β-cells^118^. It is a crucial anabolic hormone, allowing organisms to break down proteins, carbohydrates, and fats by absorbing glucose molecules^118^. Global insulin insufficiency in adult *Diemictylus viridescens* newts causes impaired limb and tail regeneration^119^. When 90% of the pancreas is destroyed, modeling a diabetes phenotype in the newt, limb, and tail amputees form sparsely populated blastemas and subsequently exhibit either abnormal or inhibited regeneration^119^. However, if the pancreas is allowed to partially regenerate before amputation, blastemal cells densely populate the wound site, and regeneration proceeds as normal^119^. Selectively destroying beta cells, instead of removing most of the pancreas, completely inhibits limb and tail regeneration^119^. It is important to acknowledge, however, that in this study, the pituitary gland was removed, which caused many other systemic effects beyond pancreas degeneration, and these secondary effects could also impact the regenerative process.

Notably, it has been shown in vitro that insulin is necessary for the mitogenic effect of innervation on newt blastemal cells^9^. In one study, without the presence of insulin in the culture medium, DNA synthesis dropped to 40% of the levels seen in innervated blastemal explants^13^. When additional insulin was added to the culture medium of innervated explants, DNA synthesis increased over 200% as compared with control samples^13^. This phenomenon has also been replicated in zebrafish, as zebrafish with phenotypes modeling type I diabetes demonstrate impaired regeneration after caudal fin amputation at time points up to 72 h post amputation^120^.

Insulin has also been implicated in wound healing and regenerative events in mammalian models. In mice, administration of insulin after optic nerve damage promotes dendrite regeneration, including at important synaptic sites, through activation of mammalian target of rapamycin 1 and 2^121^. In addition, topical insulin application after a burn injury accelerates the rate of healing in rats with type I diabetes by shortening the inflammatory phase of healing^122^. In humans, treatment of pressure ulcers with topical insulin reduces the surface area of ulcers by day seven of treatment^123^.

One of the remaining challenges in skin regeneration is regulating collagen production and formation during the repair process. Similarly to the previously discussed topical insulin experiments, another study found that the application of a hydrogel containing insulin and insulin-conjugated keratin was shown to accelerate and improve dermal wound healing during the first 2 weeks of healing^124^. Unfortunately, these topical applications are only relevant as a treatment for external injuries of the skin. Some new technologies developed to ameliorate bone regeneration in mammals use insulin as an element of a scaffolding technique^125^. The insertion of insulin-releasing micro and nanospheres into wound sites stabilizes bone implants, improves the rate of healing, and is being researched as a solution for the high rate of early implant failure^126^. Like leptin, although insulin has been very deeply studied in a variety of regenerative contexts, its most well-known function of glucose regulation might not suggest that it has such a profound effect on regenerative outcome across many contexts and organisms.

### Peptide hormones: Dilp8

*Drosophila* Insulin-Like Peptide 8 (Dilp8) is a well-researched peptide hormone implicated in *Drosophila* imaginal disc regeneration^127^. Imaginal discs are located internally in *Drosophila* larvae but develop into external adult structures during metamorphosis^128^. Regeneration of imaginal discs is epimorphic, involving local cell proliferation at the wound site to create a blastema^128^. Dilp8 functions in conjunction with the well-characterized JAK/STAT pathway, which is implicated in the early stages of injury repair in immature *Drosophila*^129^. This pathway controls both local and global injury responses and thus allows for the coordination of regenerative recovery with the developmental timeline of the organism^129^.

As a part of this coordination process, Dilp8 extends larval development, causing a developmental delay and slowing the growth of any undamaged imaginal discs^130^. This is modulated by the prothoracic gland, which activates nitric oxide synthase upon signaling from Leucine-rich repeat-containing G protein-coupled receptor 3 (Lgr3), the receptor that binds Dilp8^130^. Lgr3 is required for the Dilp8-mediated developmental delay, and animals that have Lgr3-depleted neurons show no developmental delay^130^. In addition, this Dilp8-mediated developmental delay results in a much later peak of ecdysone expression, which serves to delay metamorphosis^127^. However, there is no evidence to suggest that Dilp8-mediated signaling interacts with juvenile hormone (JH) during regenerative events^127^, despite the known function of JH as a regulator of ecdysone production and insulin release in *Drosophila*^131^. Thus, Dilp8, Lgr3, and ecdysone are important elements to consider in the context of regeneration and wound healing because they modulate both local and global responses to injury and help to coordinate injury response with development in immature organisms.

### Non-endocrine factors: molting

Similarly to the Dilp8-mediated developmental delay seen in *Drosophila*, growth and regeneration are interrelated in crabs. In two different crab species, *Hemigrapsus oregonensis* and *Pachygrapsus crassipes*, limb amputation has an effect on the size of the animal post-molt^132^. If a molting event occurs close to the time of amputation, limbs regenerate successfully, but the crabs are smaller than controls after molting^132^. In addition, crab size post-molt decreases with multiple regeneration events at or near the time of molting^132^. If the amputation event occurred within 2 weeks of the molt, a successful molt is prioritized and amputated limbs do not regenerate successfully^132^. This phenomenon has not been further investigated since the publishing of these initial results in 1975.

Regeneration events also induce a delay in molting in the cockroach^133^. Like many invertebrates, cockroaches undergo autotomy, the defensive shedding of a body part—in this case, a limb—during times of extreme stress or injury^134^. Upon autotomy, an ecdysone-mediated delay of molting occurs. The length of this delay is thought to be directly proportional to the amount of regenerating tissue^133^.

A similar phenomenon has since been investigated in *Gryllus bimaculatus*, colloquially known as the “two-spotted cricket”^135^. Cricket limbs are six-segmented, and upon amputation at the third segment, limb segments are regenerated via a series of four molts over a period of 3 days^135^. The molting process is essential to the development of the cricket. During normal development, molting allows for rapid cell division and the formation of new cuticles, which form its exoskeleton^135^. These processes are recapitulated during regeneration.

It is important to note that, in all three of these cases, the molting process that allows for regeneration mostly involves the shedding of an acellular exoskeleton, which allows for the growth of the animal. Thus, signals for new growth and patterning happen exclusively in the tissues, which lie below this exoskeleton. However, because molting is vital for successful growth, and because growth and regeneration share several characteristics, including high levels of cell proliferation, it seems likely that the signals that cue molting during growth might also cue molting during regeneration. The regeneration timelines of these organisms are restricted by the molting process, so the molting system may provide a restricted environment that allows both growth and regeneration processes to occur. It is also possible, however, that in these insect and crustacean models, the molt simply serves to reveal the regenerate, rather than directly contributing to the regenerative process.

### Non-endocrine factors: glucose

The impact of glucose on wound healing is most commonly investigated in the context of diabetes research. Animal models of diabetes are created through the generation of hyperglycemic conditions and are consequently a useful tool for analyzing the impact of glucose dysregulation on wound healing.

During early phases of wound healing, neutrophils form extracellular traps (NETs), fibrous, chromatin-containing structures that bind and destroy extracellular pathogens as a first line of defense against infection^136^; however, NET antimicrobial mechanisms can also lead to increased inflammation and tissue damage, impairing the wound-healing process^137^. Upon injury, hyperglycemic conditions, such as those present in murine diabetes models, stimulate neutrophils to produce elevated levels of extracellular traps, and this has been shown to hinder skin wound healing^138^. In vitro experiments using murine and human cells have also shown that such hyperglycemic environments inhibit fibroblast migration^139^, impairing angiogenesis^140^, and inducing oxidative stress^141^.

Elevated extracellular glucose has also been linked to wound healing impairments independently of diabetes-specific research. For example, when rat peritoneal mesothelial monolayers are treated with media containing elevated glucose levels, cell migration is reduced and fewer focal adhesions are made between cells^142^. This finding is aligned with the Warburg Effect, the phenomenon in which rapidly proliferating cells shift to anaerobic glycolysis^143^, which has been hypothesized to occur in regenerating vertebrate appendages^144^. Studies that have examined the role of the Warburg Effect on epimorphic regeneration have recently been reviewed^144,145^, although more investigation is needed to fully elucidate the mechanism and impact of the shift to anaerobic glycolysis in regenerating tissues. Furthermore, connections between a local Warburg effect and systemic processes need further investigation, as does possible system-wide Warburg effects during local repair and regeneration. These observations are also reflective of the previously discussed salt osmoregulation experiments (see “Salinity and osmoregulation” subsection), suggesting that elevated global concentrations of compounds—not limited to salts—may dampen efficient wound healing.

## Conclusion

Broad, systemic factors are critical to consider in the context of regeneration. Bringing a wide variety of species into the conversation yields important insights that may not be gleaned from more limited scopes. However, engaging in cross-species comparisons poses challenges that must continually be considered. While species may share some commonalities in their regenerative processes, all factors in the healing mechanism may not necessarily be identical. Moreover, as model organisms may be studied in different preferred regenerative contexts—for example, limbs for salamanders, antlers for deer, and hearts for zebrafish and neonatal mice—complications may arise when attempting to generalize diverse cross-system regenerative research, even when the factors investigated in cross-species studies are identical.

The existing data prompt a number of unanswered questions about systemic factors and regeneration. Certain phenomena have not yet been clarified since the 20th century, such as the relationship between growth and regeneration, as seen in molting crabs, as well as the mechanistic role of prolactin in the context of regeneration. In addition, the perhaps unexpected roles of insulin and leptin in the regenerative context allude to the possibility of other hormones acting as regenerative agonists or antagonists.

Regardless, synthesizing currently available data provide a clearer picture of systemic factors that contribute to regenerative commonalities and differences between species and systems. These comparisons may reveal information about promoters, inhibitors, and non-effectors of regeneration and repair. Among their results may be critical operational differences within the same pathway between great and poor regenerators that may be capitalized upon to stimulate and supplement regenerative processes in less regenerative organisms. Hopefully, existing and future studies can be leveraged to identify conserved pathways—as well as novel applications of pathways that might be specific to certain clades of animals—for therapeutic discovery in wound healing and regeneration.

## References

[1] Wang W (2020). Changes in regeneration-responsive enhancers shape regenerative capacities in vertebrates. Science.

[2] Poss KD (2010). Advances in understanding tissue regenerative capacity and mechanisms in animals. Nat. Rev. Genet..

[3] King BL, Yin VP (2017). Prioritizing studies on regeneration in nontraditional model organisms. Regen. Med..

[4] Simon HG (1997). A novel family of T-box genes in urodele amphibian limb development and regeneration: candidate genes involved in vertebrate forelimb/hindlimb patterning. Dev. Camb. Engl..

[5] Leigh ND (2020). von Willebrand factor D and EGF domains is an evolutionarily conserved and required feature of blastemas capable of multitissue appendage regeneration. Evol. Dev..

[6] Natarajan N (2018). Complement receptor C5aR1 plays an evolutionarily conserved role in successful cardiac regeneration. Circulation.

[7] Haller S (2017). mTORC1 activation during repeated regeneration impairs somatic stem cell maintenance. Cell Stem Cell.

[8] Cox, B. D., Yun, M. H. & Poss, K. D. Can laboratory model systems instruct human limb regeneration? *Development* **146**, dev181016 (2019). 10.1242/dev.181016 PMC6917474 31578190

[9] Vethamany-Globus S (1987). Hormone action in newt limb regeneration: insulin and endorphins. Biochem. Cell Biol. Biochim. Biol. Cell.

[10] Maier CE, Singer M (1981). The effect of prolactin on the rate of forelimb regeneration in newts exposed to photoperiod extremes. J. Exp. Zool..

[11] Schauble MK, Nentwig MR (1974). Temperature and prolactin as control factors in newt forelimb regeneration. J. Exp. Zool..

[12] Goss RJ (1969). Photoperiodic control of antler cycles in deer. I. Phase shift and frequency changes. J. Exp. Zool..

[13] Vethamany-Globus S, Globus M, Tomlinson B (1978). Neural and hormonal stimulation of DNA and protein synthesis in cultured regeneration blastemata in the newt Notophthalmus viridescens. Dev. Biol..

[14] Miao Z-F (2020). A dedicated evolutionarily conserved molecular network licenses differentiated cells to return to the cell cycle. Dev. Cell.

[15] Wan DC (2018). Honey bee Royalactin unlocks conserved pluripotency pathway in mammals. Nat. Commun..

[16] Bourque CW (2008). Central mechanisms of osmosensation and systemic osmoregulation. Nat. Rev. Neurosci..

[17] Enyedi B, Kala S, Nikolich-Zugich T, Niethammer P (2013). Tissue damage detection by osmotic surveillance. Nat. Cell Biol..

[18] Binger KJ (2015). High salt reduces the activation of IL-4– and IL-13–stimulated macrophages. J. Clin. Invest..

[19] Godwin JW, Pinto AR, Rosenthal NA (2013). Macrophages are required for adult salamander limb regeneration. Proc. Natl Acad. Sci..

[20] Eming SA, Martin P, Tomic-Canic M (2014). Wound repair and regeneration: mechanisms, signaling, and translation. Sci. Transl. Med..

[21] Lopez MLSS, Gomez RA (2010). The renin phenotype: roles and regulation in the kidney. Curr. Opin. Nephrol. Hypertens..

[22] Gomez RA, Sequeira-Lopez MLS (2018). Renin cells in homeostasis, regeneration and immune defence mechanisms. Nat. Rev. Nephrol..

[23] Hall JE (2003). Historical perspective of the renin-angiotensin system. Mol. Biotechnol..

[24] Bernasconi R, Nyström A (2018). Balance and circumstance: the renin angiotensin system in wound healing and fibrosis. Cell Signal..

[25] Prabhu SD, Frangogiannis NG (2016). The biological basis for cardiac repair after myocardial infarction: from inflammation to fibrosis. Circ. Res..

[26] Shock BC, Foran CM, Stueckle TA (2009). Effects of salinity stress on survival, metabolism, limb regeneration, and ecdysis in Uca pugnax. J. Crustac. Biol..

[27] Wang, H. et al. Transcriptomic analysis of adaptive mechanisms in response to sudden salinity drop in the mud crab, Scylla paramamosain. *BMC Genomics* **19**, 421 (2018). 10.1186/s12864-018-4803-x PMC5984308 29855258

[28] Faria, S. C., Provete, D. B., Thurman, C. L. & McNamara, J. C. Phylogenetic patterns and the adaptive evolution of osmoregulation in fiddler crabs (Brachyura, Uca). *PLoS ONE* **12**, e0171870 (2017). 10.1371/journal.pone.0171870 PMC5300755 28182764

[29] Yokoyama H (2018). Skin regeneration of amphibians: a novel model for skin regeneration as adults. Dev. Growth Differ..

[30] Johnson SL, Weston JA (1995). Temperature-sensitive mutations that cause stage-specific defects in Zebrafish fin regeneration. Genetics.

[31] Finn JP, Nielsen NO (1971). The effect of temperature variation on the inflammatory response of rainbow trout. J. Pathol..

[32] Anderson CD, Roberts RJ (1975). A comparison of the effects of temperature on wound healing in a tropical and a temperate teleost. J. Fish. Biol..

[33] Ream RA, Theriot JA, Somero GN (2003). Influences of thermal acclimation and acute temperature change on the motility of epithelial wound-healing cells (keratocytes) of tropical, temperate and Antarctic fish. J. Exp. Biol..

[34] Gillooly JF (2001). Effects of size and temperature on metabolic arte. Science.

[35] Tattersall GJ, Tyson TM, Lenchyshyn JR, Carlone RL (2012). Temperature preference during forelimb regeneration in the red-spotted newt notophthalmus viridescens: limb regrowth and thermal preference in newts. J. Exp. Zool. Part Ecol. Genet. Physiol..

[36] Hirose K (2019). Evidence for hormonal control of heart regenerative capacity during endothermy acquisition. Science.

[37] Paatela E, Munson D, Kikyo N (2019). Circadian regulation in tissue regeneration. Int. J. Mol. Sci..

[38] Borjigin J, Samantha Zhang L, Calinescu A-A (2012). Circadian regulation of pineal gland rhythmicity. Mol. Cell Endocrinol..

[39] Maier CE, Singer M (1977). The effect of light on forelimb regeneration in the newt. J. Exp. Zool..

[40] Maier CE, Singer M (1982). The effect of limiting light to the pineal on the rate of forelimb regeneration in the newt. J. Exp. Zool..

[41] Liversage RA, Stewart WE, McLaughlin DS (1984). In vitro studies of the influence of prolactin on tail regeneration in the adult newtNotophthalmus viridescens. Wilhelm. Rouxs Arch. Dev. Biol..

[42] Xue Y (2017). Modulation of circadian rhythms affects corneal epithelium renewal and repair in mice. Investig. Opthalmology Vis. Sci..

[43] Deshayes N, Genty G, Berthelot F, Paris M (2018). Human long-term deregulated circadian rhythm alters regenerative properties of skin and hair precursor cells. Eur. J. Dermatol. EJD.

[44] Stokes K (2017). The circadian clock gene BMAL1 coordinates intestinal regeneration. Cell Mol. Gastroenterol. Hepatol..

[45] Hoyle NP (2017). Circadian actin dynamics drive rhythmic fibroblast mobilization during wound healing. Sci. Transl. Med..

[46] Khapre RV (2014). BMAL1-dependent regulation of the mTOR signaling pathway delays aging. Aging.

[47] Zagni C (2017). PTEN mediates activation of core clock protein BMAL1 and accumulation of epidermal stem cells. Stem Cell Rep..

[48] Kowalska E (2013). NONO couples the circadian clock to the cell cycle. Proc. Natl Acad. Sci..

[49] Schauble MK (1972). Seasonal variation of newt forelimb regeneration under controlled environmental conditions. J. Exp. Zool..

[50] Schauble MK, Tyler DB (1972). The effect of prolactin on the seasonal cyclicity of newt forelimb regeneration. J. Exp. Zool..

[51] Goss RJ (1995). Future directions in antler research. Anat. Rec..

[52] Li C, Zhao H, Liu Z, McMahon C (2014). Deer antler – a novel model for studying organ regeneration in mammals. Int. J. Biochem. Cell Biol..

[53] Goss RJ (1961). Experimental investigation of morphogenesis in the growing antler. J. Embryol. Exp. Morphol..

[54] Goss RJ (1980). Photoperiodic control of antler cycles in deer. V. Reversed seasons. J. Exp. Zool..

[55] Shi Z, Barrell G (1994). Thyroid hormones are required for the expression of seasonal changes in red deer (Cervus elaphus) stags. Reprod. Fertil. Dev..

[56] Suttie JM, Lincoln GA, Kay RNB (1984). Endocrine control of antler growth in red deer stags. Reproduction.

[57] Faucheux C (2004). Recapitulation of the parathyroid hormone-related peptide-Indian hedgehog pathway in the regenerating deer antler. Dev. Dyn..

[58] Akhtar RW (2019). Identification of proteins that mediate the role of androgens in antler regeneration using label free proteomics in sika deer (Cervus nippon). Gen. Comp. Endocrinol..

[59] Dong Z, Coates D, Liu Q, Sun H, Li C (2019). Quantitative proteomic analysis of deer antler stem cells as a model of mammalian organ regeneration. J. Proteom..

[60] Gudernatsch JF (1912). Feeding experiments on tadpoles: I. The influence of specific organs given as food on growth and differentiation. A contribution to the knowledge of organs with internal secretion. Arch. F.ür. Entwicklungsmechanik Org..

[61] Larras-Regard E, Taurog A, Dorris M (1981). Plasma T4 and T3 levels in Ambystoma tigrinum at various stages of metamorphosis. Gen. Comp. Endocrinol..

[62] Young HE, Bailey CF, Dalley BK (1983). Envirnmental conditions prerequisite for complete limb regeneration in the postmetamorphic adult land-phase salamander, Ambystoma. Anat. Rec..

[63] Monaghan JR (2014). Experimentally induced metamorphosis in axolotls reduces regenerative rate and fidelity: Axolotl Metamorphosis reduces. Regeneration.

[64] Dent JN (1962). Limb regeneration in larvae and metamorphosing individuals of the South African clawed toad. J. Morphol..

[65] Barakat-Walter I, Kraftsik R (2018). Stimulating effect of thyroid hormones in peripheral nerve regeneration: research history and future direction toward clinical therapy. Neural Regen. Res..

[66] Bonett RM, Hoopfer ED, Denver RJ (2010). Molecular mechanisms of corticosteroid synergy with thyroid hormone during tadpole metamorphosis. Gen. Comp. Endocrinol..

[67] Marshall LN (2019). Stage-dependent cardiac regeneration in Xenopus is regulated by thyroid hormone availability. Proc. Natl. Acad. Sci..

[68] Simon A, Tanaka EM (2013). Limb regeneration: limb regeneration. Wiley Interdiscip. Rev. Dev. Biol..

[69] Ekmektzoglou KA (2006). A concomitant review of the effects of diabetes mellitus and hypothyroidism in wound healing. World J. Gastroenterol..

[70] Grunfeld R, Kunselman A, Bustillo J, Juliano PJ (2011). Wound complications in thyroxine-supplemented patients following foot and ankle surgery. Foot Ankle Int..

[71] Kromuszczyńska J, Kołodziej Ł, Jurewicz A (2018). Wound healing complications in patients with and without systemic diseases following hallux valgus surgery. PloS ONE.

[72] Kranz D, Hecht A, Fuhrmann I (1976). The influence of hyperthyroidism and hypothyroidism on the wound healing of experimental myocardial infarction in the rat. Exp. Pathol. (Jena.).

[73] Safer JD, Crawford TM, Holick MF (2004). A role for thyroid hormone in wound healing through keratin gene expression. Endocrinology.

[74] Schaaf MJM, De Kloet ER, Vreugdenhil E (2000). Corticosterone effects on BDNF expression in the hippocampus implications for memory formation. Stress.

[75] Quax RA (2013). Glucocorticoid sensitivity in health and disease. Nat. Rev. Endocrinol..

[76] Lewis JL, Sullivan AM (2020). Salamander stress and duress: the relationship between CORT, autotomy and regeneration, and exploratory behaviour. Zool. Jena. Ger..

[77] Thomas JR, Woodley SK (2015). Treatment with corticosterone delays cutaneous wound healing in male and female salamanders. Gen. Comp. Endocrinol..

[78] Pianca, N. et al. Glucocorticoid Receptor ablation promotes cardiac regeneration by hampering cardiomyocyte terminal differentiation. 10.1101/2020.01.15.901249 (2020).

[79] Cutie S, Payumo AY, Lunn D, Huang GN (2020). In vitro and in vivo roles of glucocorticoid and vitamin D receptors in the control of neonatal cardiomyocyte proliferative potential. J. Mol. Cell Cardiol..

[80] Raff H (2016). CORT, Cort, B, corticosterone, and now cortistatin: enough already!. Endocrinology.

[81] Nesan D, Vijayan MM (2013). Role of glucocorticoid in developmental programming: evidence from zebrafish. Gen. Comp. Endocrinol..

[82] Hartig EI, Zhu S, King BL, Coffman JA (2016). Cortisol-treated zebrafish embryos develop into pro-inflammatory adults with aberrant immune gene regulation. Biol. Open.

[83] Brufani M (2017). Novel locally active estrogens accelerate cutaneous wound healing-part 2. Sci. Rep..

[84] Mukai K, Urai T, Asano K, Nakajima Y, Nakatani T (2016). Evaluation of effects of topical estradiol benzoate application on cutaneous wound healing in ovariectomized female mice. PLoS ONE.

[85] Campbell L (2010). Estrogen promotes cutaneous wound healing via estrogen receptor beta independent of its antiinflammatory activities. J. Exp. Med..

[86] Ikeda K, Horie-Inoue K, Inoue S (2019). Functions of estrogen and estrogen receptor signaling on skeletal muscle. J. Steroid Biochem. Mol. Biol..

[87] Lee W-J, Lee S-C, Lee J-H, Rho G-J, Lee S-L (2016). Differential regulation of senescence and in vitro differentiation by 17β-estradiol between mesenchymal stem cells derived from male and female mini-pigs. J. Vet. Sci..

[88] Xu, K. et al. Effects of Bakuchiol on chondrocyte proliferation via the PI3K‐Akt and ERK1/2 pathways mediated by the estrogen receptor for promotion of the regeneration of knee articular cartilage defects. *Cell Prolif*. **52**, e12666 (2019). 10.1111/cpr.12666 PMC6797515 31407423

[89] Batmunkh B (2017). Estrogen accelerates cell proliferation through estrogen receptor α during rat liver regeneration after partial hepatectomy. Acta Histochem. Cytochem..

[90] Kao T-L (2018). Estrogen receptors orchestrate cell growth and differentiation to facilitate liver regeneration. Theranostics.

[91] Tsugawa Y, Natori M, Handa H, Imai T (2019). Estradiol accelerates liver regeneration through estrogen receptor α. Clin. Exp. Gastroenterol..

[92] Xu S (2020). Estrogen accelerates heart regeneration by promoting the inflammatory response in zebrafish. J. Endocrinol..

[93] Nachtrab G, Czerwinski M, Poss KD (2011). Sexually dimorphic fin regeneration in zebrafish controlled by androgen/GSK3 signaling. Curr. Biol..

[94] Ashcroft GS, Mills SJ (2002). Androgen receptor–mediated inhibition of cutaneous wound healing. J. Clin. Invest..

[95] Bird MD, Karavitis J, Kovacs EJ (2008). Sex differences and estrogen modulation of the cellular immune response after injury. Cell Immunol..

[96] Gilliver SC, Ashcroft GS (2007). Sex steroids and cutaneous wound healing: the contrasting influences of estrogens and androgens. Climacteric.

[97] Mihai MC (2019). Mechanism of 17β-estradiol stimulated integration of human mesenchymal stem cells in heart tissue. J. Mol. Cell Cardiol..

[98] Horng H-C (2017). Estrogen effects on wound healing. Int. J. Mol. Sci..

[99] Gunamalai V, Kirubagaran R, Subramoniam T (2004). Hormonal coordination of molting and female reproduction by ecdysteroids in the mole crab Emerita asiatica (Milne Edwards). Gen. Comp. Endocrinol..

[100] Hopkins PM (1989). Ecdysteroids and regeneration in the fiddler crab Uca pugilator. J. Exp. Zool..

[101] Das S, Durica DS (2013). Ecdysteroid receptor signaling disruption obstructs blastemal cell proliferation during limb regeneration in the fiddler crab, Uca pugilator. Mol. Cell Endocrinol..

[102] Jaszczak JS, Wolpe JB, Dao AQ, Halme A (2015). Nitric oxide synthase regulates growth coordination during drosophila melanogaster imaginal disc regeneration. Genetics.

[103] Narbonne-Reveau K, Maurange C (2019). Developmental regulation of regenerative potential in Drosophila by ecdysone through a bistable loop of ZBTB transcription factors. PLoS Biol..

[104] Dinan L, Lafont R (2006). Effects and applications of arthropod steroid hormones (ecdysteroids) in mammals. J. Endocrinol..

[105] Lin Y (2019). Royal jelly-derived proteins enhance proliferation and migration of human epidermal keratinocytes in an in vitro scratch wound model. BMC Complement. Altern. Med..

[106] Yakoot M, Abdelatif M, Helmy S (2019). Efficacy of a new local limb salvage treatment for limb-threatening diabetic foot wounds - a randomized controlled study. Diabetes Metab. Syndr. Obes. Targets Ther..

[107] Zhang Y, Chua S (2017). Leptin function and regulation. Compr. Physiol..

[108] Zhou BO, Yue R, Murphy MM, Peyer JG, Morrison SJ (2014). Leptin-receptor-expressing mesenchymal stromal cells represent the main source of bone formed by adult bone marrow. Cell Stem Cell.

[109] Onger ME (2017). Possible promoting effects of melatonin, leptin and alcar on regeneration of the sciatic nerve. J. Chem. Neuroanat..

[110] Cilekar M, Uysal O, Bal C, Turel S, Yılmaz S (2013). Leptin increases mitotic index and regeneration ratio in hepatectomized rats. Med. Sci. Monit. Basic Res..

[111] Poeggeler B (2010). Leptin and the skin: a new frontier. Exp. Dermatol..

[112] Kang J (2016). Modulation of tissue repair by regeneration enhancer elements. Nature.

[113] Bryant DM (2017). A tissue-mapped axolotl de novo transcriptome enables identification of limb regeneration factors. Cell Rep..

[114] Frank S, Stallmeyer B, Kämpfer H, Kolb N, Pfeilschifter J (2000). Leptin enhances wound re-epithelialization and constitutes a direct function of leptin in skin repair. J. Clin. Invest..

[115] Chablais F, Jazwinska A (2010). IGF signaling between blastema and wound epidermis is required for fin regeneration. Dev. Camb. Engl..

[116] Sun H, Lin C-H, Smith ME (2011). Growth hormone promotes hair cell regeneration in the zebrafish (Danio rerio) inner ear following acoustic trauma. PloS ONE.

[117] Huang, Y. et al. Igf signaling is required for cardiomyocyte proliferation during zebrafish heart development and regeneration. *PLoS ONE* **8**, e67266 (2013). 10.1371/journal.pone.0067266 PMC3694143 23840646

[118] Heller S, Kozlovski P, Kurtzhals P (2007). Insulin’s 85th anniversary-an enduring medical miracle. Diabetes Res. Clin. Pract..

[119] Vethamany-Globus S, Liversage RA (1973). The relationship between the anterior pituitary gland and the pancreas in tail regeneration of the adult newt. J. Embryol. Exp. Morphol..

[120] Olsen AS, Sarras MP, Intine RV (2010). Limb regeneration is impaired in an adult zebrafish model of diabetes mellitus. Wound Repair Regen..

[121] Agostinone J (2018). Insulin signalling promotes dendrite and synapse regeneration and restores circuit function after axonal injury. Brain.

[122] Azevedo FF (2019). Topical insulin modulates inflammatory and proliferative phases of burn-wound healing in diabetes-induced rats. Biol. Res. Nurs..

[123] Stephen S, Agnihotri M, Kaur S (2016). A randomized, controlled trial to assess the effect of topical insulin versus normal saline in pressure ulcer healing. Ostomy Wound Manag..

[124] Li W (2019). Synthesis and fabrication of a keratin-conjugated insulin hydrogel for the enhancement of wound healing. Colloids Surf. B Biointerfaces.

[125] Wang X (2018). Enhanced bone regeneration using an insulin-loaded nano-hydroxyapatite/collagen/PLGA composite scaffold. Int. J. Nanomed..

[126] Wang X (2019). Uniform-sized insulin-loaded PLGA microspheres for improved early-stage peri-implant bone regeneration. Drug Deliv..

[127] Colombani J, Andersen DS, Léopold P (2012). Secreted peptide Dilp8 coordinates Drosophila tissue growth with developmental timing. Science.

[128] Mattila J, Omelyanchuk L, Nokkala S (2004). Dynamics of decapentaplegic expression during regeneration of the Drosophila melanogaster wing imaginal disc. Int. J. Dev. Biol..

[129] Katsuyama T, Comoglio F, Seimiya M, Cabuy E, Paro R (2015). During Drosophila disc regeneration, JAK/STAT coordinates cell proliferation with Dilp8-mediated developmental delay. Proc. Natl Acad. Sci. UsA.

[130] Jaszczak JS, Wolpe JB, Bhandari R, Jaszczak RG, Halme A (2016). Growth coordination during drosophila melanogaster imaginal disc regeneration is mediated by signaling through the relaxin receptor Lgr3 in the Prothoracic Gland. Genetics.

[131] Mirth CK (2014). Juvenile hormone regulates body size and perturbs insulin signaling in Drosophila. Proc. Natl Acad. Sci. Usa.

[132] Kuris AM, Mager M (1975). Effect of limb regeneration on size increase at molt of the shore crabs Hemigrapsus oregonensis and Pachygrapsus crassipes. J. Exp. Zool..

[133] Kunkel JG (1977). Cockroach molting. ii. the nature of regeneration-induced delay of molting hormone secretion. Biol. Bull..

[134] Fleming PA, Muller D, Bateman PW (2007). Leave it all behind: a taxonomic perspective of autotomy in invertebrates. Biol. Rev. Camb. Philos. Soc..

[135] Hamada Y (2015). Leg regeneration is epigenetically regulated by histone H3K27 methylation in the cricket Gryllus bimaculatus. Development.

[136] Brinkmann V (2004). Neutrophil extracellular traps kill bacteria. Science.

[137] Saffarzadeh M (2012). Neutrophil extracellular traps directly induce epithelial and endothelial cell death: a predominant role of histones. PLoS ONE.

[138] Wong SL (2015). Diabetes primes neutrophils to undergo NETosis, which impairs wound healing. Nat. Med..

[139] Xuan YH (2014). High-glucose inhibits human fibroblast cell migration in wound healing via repression of bFGF-regulating JNK phosphorylation. PLoS ONE.

[140] Hu SC-S, Lan C-CE (2016). High-glucose environment disturbs the physiologic functions of keratinocytes: focusing on diabetic wound healing. J. Dermatol. Sci..

[141] Kido D (2017). Impact of diabetes on gingival wound healing via oxidative stress. PLoS ONE.

[142] Tamura M (2003). High glucose levels inhibit focal adhesion kinase-mediated wound healing of rat peritoneal mesothelial cells. Kidney Int..

[143] Vander Heiden MG, Cantley LC, Thompson CB (2009). Understanding the Warburg effect: the metabolic requirements of cell proliferation. Science.

[144] Love NR, Ziegler M, Chen Y, Amaya E (2014). Carbohydrate metabolism during vertebrate appendage regeneration: what is its role? How is it regulated?: A postulation that regenerating vertebrate appendages facilitate glycolytic and pentose phosphate pathways to fuel macromolecule biosynthesis. BioEssays.

[145] Wong AY, Whited JL (2020). Parallels between wound healing, epimorphic regeneration and solid tumors. Development.

